# Quantifying the stiffness of lumbar erector spinae during different positions among participants with chronic low back pain

**DOI:** 10.1371/journal.pone.0270286

**Published:** 2022-06-24

**Authors:** Yapeng Li, Jiafeng Yu, Jun Zhang, Zhijie Zhang, Xueqiang Wang

**Affiliations:** 1 Rehabilitation Therapy Center, Luoyang Orthopedic Hospital of Henan Province, Orthopedic Hospital of Henan Province, Luoyang, China; 2 Department of Rehabilitation Medicine, The First Affiliated Hospital of Zhengzhou University, Zhengzhou, China; 3 Department of Rehabilitation, Institute of Rehabilitation and Health Care, Hunan Traditional Chinese Medical College, Hunan, China; 4 Department of Sport Rehabilitation, Shanghai University of Sport, Shanghai, China; Mugla Sitki Kocman Universitesi, TURKEY

## Abstract

**Objective:**

The purposes of this study were to (1) detect the intra- and inter-reliabilities of the lumbar erector spinae stiffness by MyotonPRO among participants with chronic lower back pain (CLBP); (2) compare the muscle stiffness between pain and non-pain sides during different positions; (3) explore the correlation between intensity of pain and muscle stiffness.

**Design:**

Twenty participants with CLBP were recruited and the stiffness measurements were carried out by two experienced physiotherapists (operators Ⅰ and Ⅱ). Each participant was evaluated by the operatorⅠ in different postures (static prone and sitting). After a 5-day interval, the same participant was reassessed by the operatorⅠ in the static prone posture. For the inter-rater reliability test, each participant was quantified by both operators once, with 30 minutes between the measurements on the same day. The intensity of pain was evaluated using a 0–10 visual analog scale (VAS).

**Results:**

The intra- and inter-rater reliabilities were excellent (ICC = 0.88–0.99). The MDC values ranged from 25.03 to 86.26 N/m. Examples of Bland-Altman plots showed good agreement. The erector spinae stiffness on the painful sides was higher with a marked increase in the sitting position (P < .05) when compared with the non-painful side. However, there was no significant difference in the prone position (P > .05). The intensity of pain among adults with CLBP was not associated with muscle stiffness of the lumbar erector spinae muscle.

**Conclusions:**

Our findings indicated that the MyotonPRO is a feasible device in quantifying the stiffness of the lumbar erector spinae muscle in patients with CLBP. Meanwhile, the erector spinae stiffness on the painful sides was higher in the sitting when compared with the non-painful side.

## Introduction

Chronic low back pain (CLBP) is prevalent musculoskeletal discomfort and the principal cause of a decreased quality of life and disability worldwide [1]. From 1999 to 2016, its prevalence was reported to have increased 23.5% in China [2]. CLBP is significantly associated with the lumbar muscles [3–5]. Various lumbar muscles contribute to maintaining the stability of the low back during trunk movement. A recent cross-sectional study indicated that muscle degeneration, fat infiltration, alterations in muscle fiber type, and altered muscle activity, compromises proper biomechanics and motion of the spinal units in patients with CLBP [6]. Many researches have focused on identifying impaired morphology and function of spinal musculature using B-mode ultrasound [7] and electromyography (EMG) [8]. However, EMG can provide only significant parameters reflecting the muscle fatigue and activation [7]. Furthermore, external environmental noise, sweating, and non-target muscle tissue also interfere with the EMG signal [9]. B-mode ultrasound can provide only the morphology of the target muscle (i.e., thickness or cross-sectional area).

Biomechanical characteristics of muscles such as stiffness are objective parameters that manifest tissue condition [10] and are references for clinicians to use to diagnose and treat. The accurate modulation of lumbar erector spinae muscle stiffness is unclear. It is therefore important to explore biomechanical characteristics of lumbar muscles for further detecting the mechanism of CLBP. To date, there is no sufficient study to investigate the lumbar muscle stiffness changes like other mechanical muscle properties in CLBP populations.

Many new technologies such as magnetic resonance elastography (MRE) [11] and shear wave elastography (SWE) [12] have been applied to quantify the biomechanical characteristics of muscles. However, there are certain limitations. MRE requires large mechanical vibrators. Moreover, it has inherent limitations due to complex methodological operations, acquisition durations, transportability, accessibility, and cost efficiency. SWE can be used to assess lumbar muscle stiffness, [12] but it is expensive and not available in most clinics. Therefore, a more convenient assessment technology is indispensable to quantify lumbar muscle stiffness. MyotonPRO is a hand-held, non-invasive device to evaluate muscles stiffness [13, 14]. Feng et al. [15] has demonstrated that the stiffness of gastrocnemius muscle belly and Achilles tendon measured by MyotonPRO is related to the Young’s modulus of those quantified by SWE. More recently, the MyotonPRO has been reported to quantify the lumbar muscles and myofascia among healthy populations [10, 16].

Furthermore, poor posture might be an occupational risk factor for CLBP. Alessa and Ning [17] reported that lumbar muscle activities were influenced during trunk bending. Prolonged standing also leads to low back discomfort [18]. Interestingly, Porter and Gyi [19] found that prolonged sitting increases the risk of low back pain. In Gsell et al. [3] study, ectopic calcification of the spine causes an inverse compensatory change, i.e., muscle fiber elastic modulus reduces in the adjacent paraspinal muscles. Anatomically, intervertebral disc degeneration causes an increase in the passive stiffness of lumbar erector spinae. The lack of movement and long-term poor sitting contribute to the occurrence of hyperirritable spots in skeletal muscles with a specific referred pain pattern, namely myofascial pain syndrome. This spots with a specific referred pain pattern may be more easily provoked in a sitting position. In our previous study [20], we examine changes in the lumbar erector spinae muscles stiffness in healthy subjects while individuals adopted the different posture (the static prone, sitting and upright standing posture). Results demonstrate there were no significant difference in different posture between left and right sides. The stiffness in different posture was as follows: sitting > the static prone > upright standing. These findings indicate that it is meaningful to explore the modulation of lumbar erector spinae muscle stiffness in different postures to help prevent CLBP. To date, there is a lack of data about assessing the lumbar muscles stiffness in pathological conditions in posture from prone to sitting, and it is therefore necessary to quantify lumbar stiffness by MyotonPRO in subjects with CLBP, providing a reference for clinicians to diagnose CLBP and improve the rehabilitation therapy.

The objectives of this study were to (1) detect the intra- and inter-reliabilities of the lumbar erector spinae stiffness by MyotonPRO among adults with CLBP; (2) compare the stiffness of lumbar erector spinae between pain and non-pain sides and the changes of stiffness from prone to sitting; (3) explore the correlation between intensity of pain and lumbar erector spinae stiffness.

## Materials and methods

The study was conducted after receiving approval from the ethics committee of Henan Provincial Luoyang Orthopedic Hospital and in accordance with the Helsinki Declaration (KY2019-001-01). This study was registered in International Clinical Trials Registry Platform (ChiCTR1900025471). All subjects provided written informed consent.

According to the minimum sample size calculation method established in the study of Walter et al. [21], the acceptable minimum ICC value (ρ_0_) in this study was 0.5, and the expected ICC value (ρ_1_) was 0.8. Based on α = 0.05, β = 0.20, an 80% power, and 3 replicate measurements, the minimum required sample size is 15. Twenty volunteers with CLBP between the ages of 18 and 77 years were recruited to participate in this study. All the candidates were hospitalized patients with diagnosis-confirmed by an experienced orthopaedic surgeon. The participants were recruited from Henan Provincial Luoyang Orthopedic Hospital between May and June 2019. In the experiments, every participant had a unique code that could identify individual participants during or after data collection. The inclusion criteria were participants over 18 years of age with unilateral CLBP (except for bilateral or central CLBP) lasting 3 months or more [22]. Unilateral CLBP was defined on the basis of patient report of pain location on one side of the back with or without sciatica [23]. The exclusion criteria were a history of previous spine surgery, spinal deformity (i.e., scoliosis, kyphosis), osteoporosis, lumbar disc protrusion, and a body mass index (BMI) of 30 kg/m^2^ or greater [24].

The stiffness of the lumbar erector spinae muscles was assessed using a small, noninvasive hand-held MyotonPRO device (Myoton Ltd., Estonia). The method of measurement is based on recording damped natural oscillation of soft tissue in the form of an acceleration signal. First, the probe of the MyotonPRO is positioned perpendicular and stable on the skin surface of the belly of the lumbar erector spinae muscle. Second, the preloaded pressure (0.18 N) was applied to slightly compress subcutaneous superficial tissue, and a mechanical impulse was released quickly (0.4N), causing muscle tissue damped oscillation. The signal of the oscillation pattern recorded by the accelerometer was used to calculate the muscle stiffness (N/m). The calculation formula is: S = *a*_*max*_ ∙ *m*_*probe*_/Δ*l*, where *a*_*max*_ is the acceleration of the damped oscillation, *m*_*probe*_ is the mass of the measurement mechanism, and Δ*l* is the maximal displacement of the tissue.

**Fig 1:**
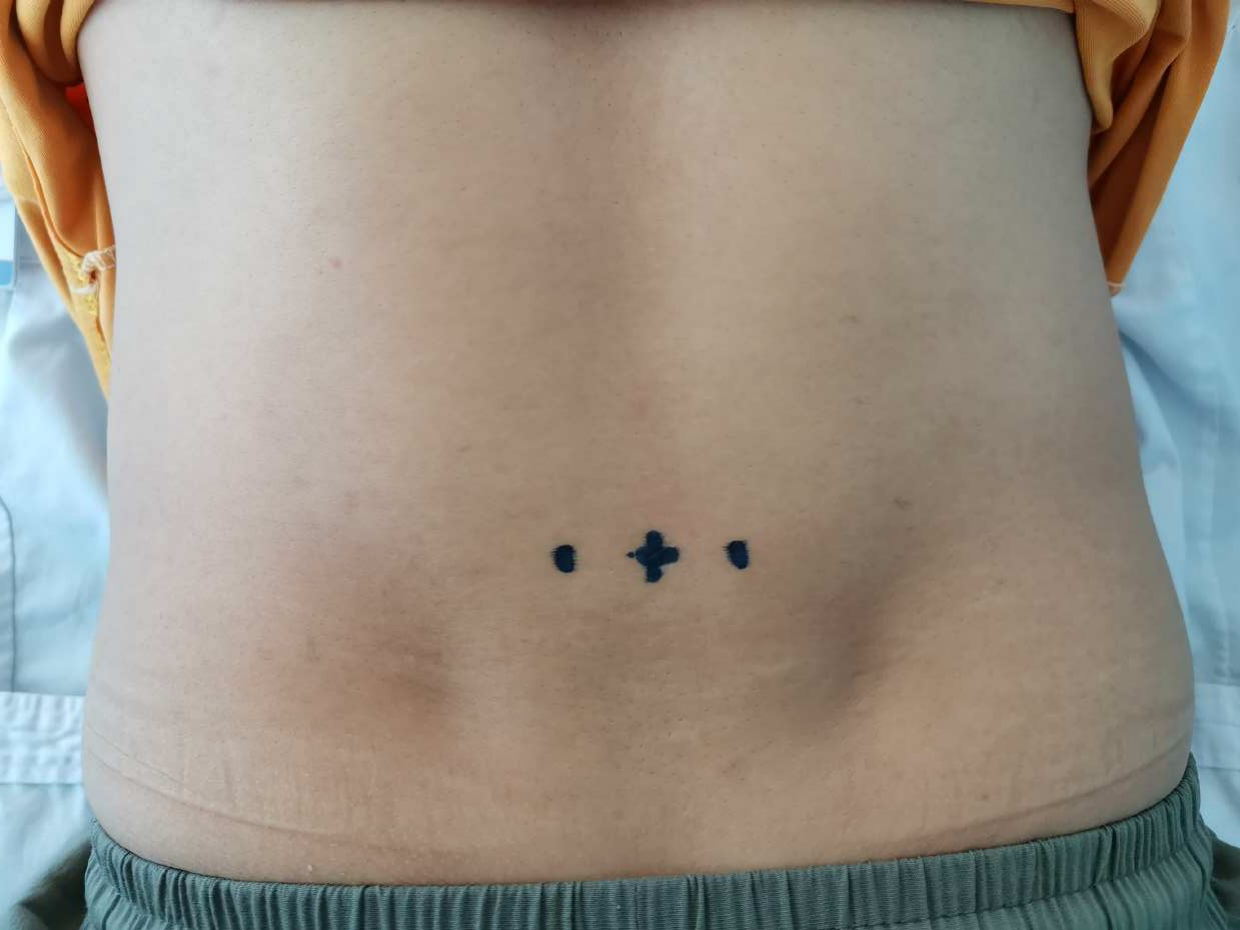
The measurement sites of the erector spinae stiffness.

Measurements were taken in the Rehabilitation Therapy Center in our hospital. The measurement sites were defined as the palpable muscle belly one-finger breadth from the spinous process at level L_4_ (Fig 1) [10, 16, 24]. Hu et al. [25] reported that standard error of measurement stiffness at L_4_ were lower than those at other levels. First, stiffness data were acquired in a prone position on an examination bed with arms resting at the side and the ankle in a relaxed position. Second, the subject was asked to sit upright on a stool with the head in neutral position. In the process of testing, both operators did not know which side of the participant was painful. Moreover, we asked all participants to hold their breath for 5 seconds at the end of inspiration to minimize the confounding factor resulting from changes of intra-abdomen pressure occurring with natural respiratory cycles. The muscle stiffness was measured three times, and the average of the three measured values was taken. The room temperature was held constant at 25°C.

The stiffness measurements were carried out by two experienced physiotherapists (YPL and ZJZ). Both operators had used MyotonPRO as part of daily scientific research for 2–3 years and practiced using the device on the lumbar erector spinae muscle prior to the start of the investigation. To assess the intra-rater reliability of the erector spinae stiffness-related measurements using the MyotonPRO, repeated measurements were obtained by operator YPL according to the above protocol. Two measurements were taken from the same participants each day in 5-day intervals, and the average of these two measures was used for intra-rater reliability calculations. For the inter-rater reliability test, each participant was quantified by both operators (YPL and ZJZ) once, with 30 minutes between the measurements on the same day. The mean of three measurements was used for statistical analysis.

The intensity of pain was evaluated using a 0–10 visual analog scale (VAS), with 0 meaning no pain and 10 meaning worst pain [26].

### Statistical analysis

Statistical analyses were performed using SPSS 16.0 software (SPSS Inc, Chicago, IL). Descriptive statistics were used to summarize all demographic data as means and standard deviations (SDs). Intraclass correlation coefficients (ICCs) with 95% confidence interval (CI) were used to assess intra-rater within-session reliability (one-way random model) and between-rater reliability (two-way random model). For intra-rater reliability, the association within session was analyzed using a one-way random model consistency. Inter-rater reliability was analyzed using a two-way random model consistency. The following classification was used to interpret reliability: excellent = 0.90–1.00; good = 0.70–0.89; moderate = 0.50–0.69; poor < 0.49 [27]. The standard error measurement (SEM) was computed using the formula SEM = standard deviation ×√1-ICC, and minimal detectable change was calculated using the formula MDC = 1.96×SEM ×√2. SEM% was defined as SEM% = (SEM/mean) × 100. MDC% was defined as MDC% = (MDC/mean) × 100. Bland-Altman plots were also used to provide a visual representation of degree of agreement. An independent *t* test was conducted to compare the erector spinae stiffness between the painful side and non-painful side in different postures and the changes of stiffness from prone to sitting in the painful and non-painful sides. Spearman rank correlation tests were used to examine the VAS and the erector spinae stiffness on the painful side.

## Results

Two experienced physiotherapists were recruited for the study. All participants (N = 20; mean age: 45.8 ± 18.5 years; mean height: 168.7 ± 7.9 cm; mean body mass: 69.6 ± 11.6 kg) completed the experimental protocol with no reports of medical problems, discomfort, or adverse reactions related to the study.

A summary of the results for intra- and inter-rater reliability of the erector spinae stiffness using the MyotonPRO is shown in Table 1. For intra-rater reliability, the ICC with 95% CI was 0.88 (0.55–0.97) for left, SEM = 31.12 N/m, and MDC = 86.26 N/m. The ICC with 95%CI was 0.91 (0.67–0.98) for right, SEM = 26.35 N/m, and MDC = 73.04 N/m. All SEM% and MDC% were less than 2.69% and 7.46%, respectively. For inter-rater reliability, the ICC with 95% CI was 0.99 (0.94–1.00) for left, SEM = 9.03 N/m, and MDC = 25.03 N/m. The ICC with 95%CI was 0.99 (0.94–1.00) for right, SEM = 9.64 N/m, and MDC = 26.72 N/m. All SEM% and MDC% were less than 9.14% and 25.35%, respectively.

**Table 1:** The intra and inter-operator reliability of MyotonPRO in measurement of the lumbar erector spinae muscles stiffness (N/m).

	Intra-operator reliability (mean±SD)						Inter-operrator reliability (mean±SD)					
	Test1	Test2	SEM	ICC	95%CI	MDC	OperatorⅠ	OperatorⅡ	SEM	ICC	95%CI	MDC
Left	340.33±90.25	369.93±89.85	31.12	0.88	0.55–0.97	86.26	340.33±90.25	350.50±92.28	9.03	0.99	0.94–1.00	25.03
Right	367.30±110.54	380.40±87.82	26.35	0.91	0.67–0.98	73.04	367.30±110.54	358.27±96.40	9.64	0.99	0.94–1.00	26.72

SD, standard deviation; SEM, standard error mean; ICC, intraclass correlation coefficient; MDC, minimum detectable change. 95%CI, 95% confidence interval; S, Dynamic Stiffness.

**Fig 2:**
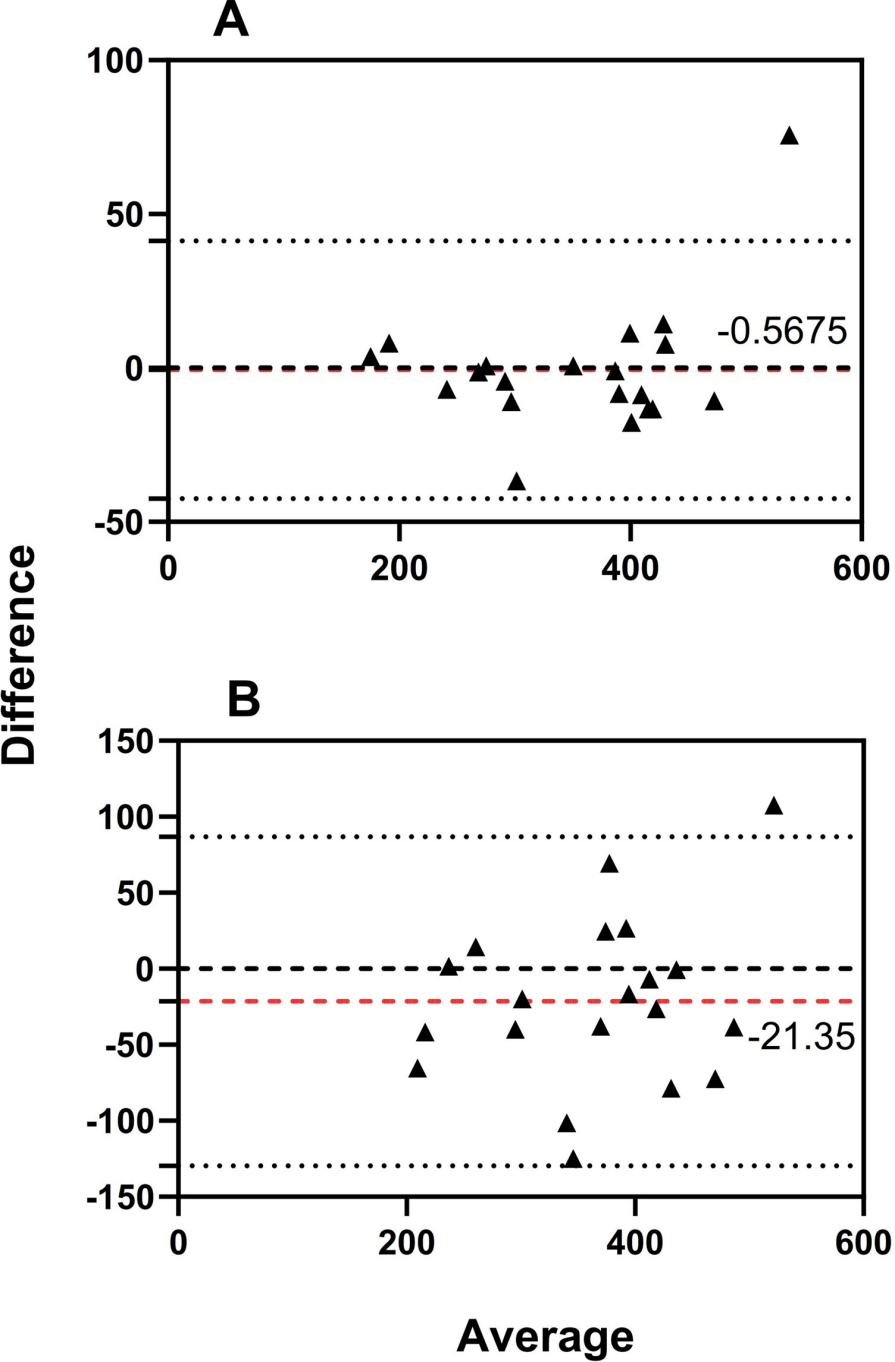
Bias of measurement between different operators (Ⅰ and Ⅱ) and limits of agreement are represented by a red dotted and two black solid lines respectively. A: Bland-Altman plot of the inter-rater reliability for the erector spinae stiffness. B: Bland-Altman plot of the intra-rater reliability for the erector spinae stiffness.

The Bland-Altman plot for the erector spinae stiffness is shown in Fig 2. For inter-rater reliability, the bias line between operator Ⅰ and operator Ⅱ was -0.5675 N/m, and the 95% limit of agreement was -42.39 to 41.26 N/m (Fig 2A). For intra-rater reliability, the bias line between test 1 and test 2 was -21.35 N/m, and the 95% limit of agreement was -129.70 to 87.00 N/m (Fig 2B).

**Fig 3:**
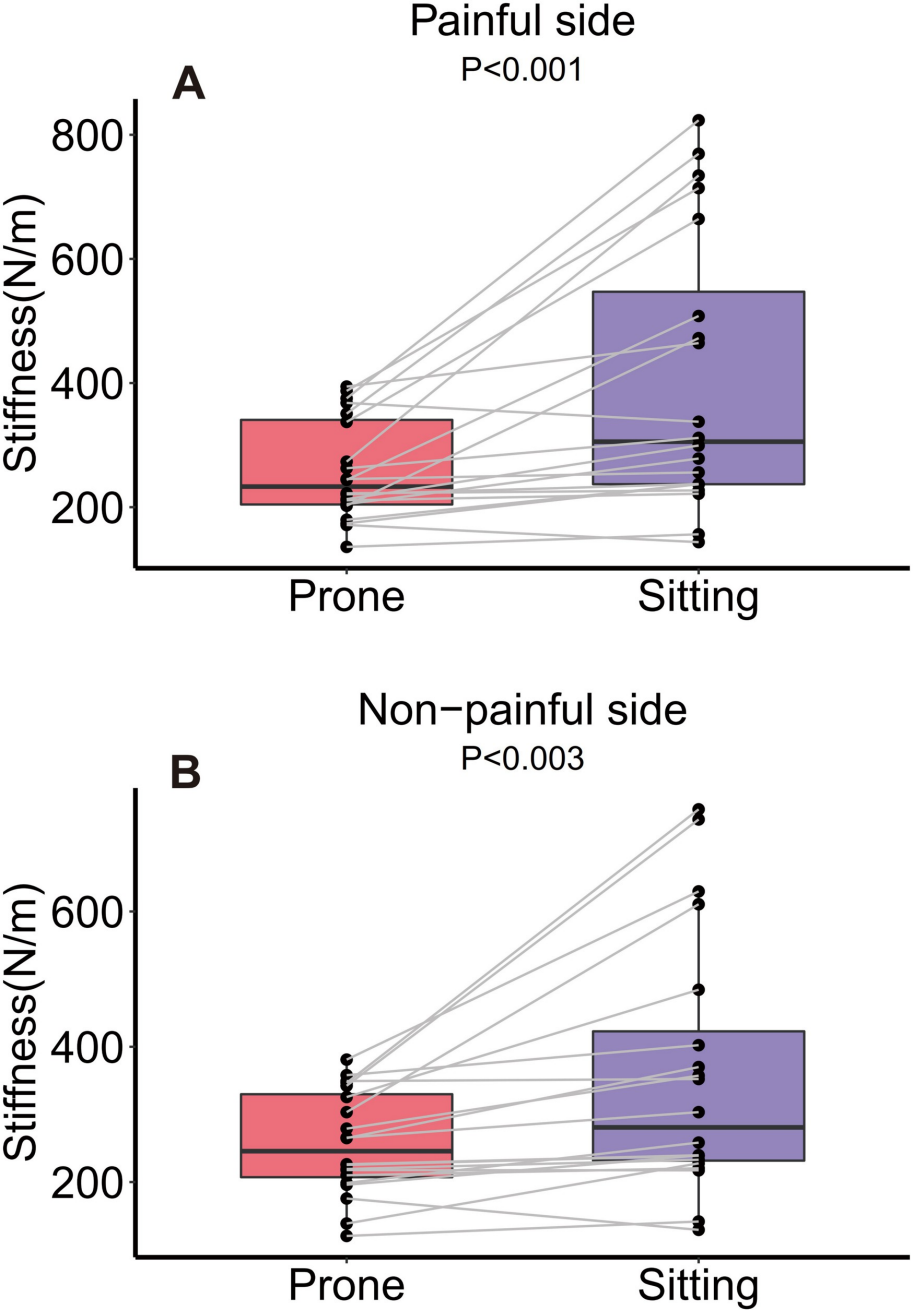
Changes in stiffness of the erector spinae muscle between the painful and non-painful sides from prone to sitting (*N* = 20). Values are mean ± SD.

In subjects with unilateral CLBP, the stiffness index measured by MyotonPRO showed significant differences between the painful and non-painful sides in sitting (*P* < .01) compared with prone (*P* > .05) positions (Table 2). Muscle stiffness increased sharply at the painful sides from prone to sitting (from 258.68 to 404.65 N/m, *P* < .01, an increase of 56.43%). On the non-painful sides, muscle stiffness increased slowly (from 257.20 to 357.13 N/m, *P* < .01, an increase of 38.85%) (Fig 3).

**Table 2:** An independent t test of the lumbar erector spinae muscles stiffness between painful and non-painful sides in human participants with CLBP.

	Prone	Sitting	Percent changes	*P*
Painful side	258.68±81.13	404.65±222.46	56.43%	.001
Non-painful side	257.20±76.24	357.13±189.00	38.85%	.003
*P*	.873	.002		

CLBP: chronic low back pain.

**Fig 4:**
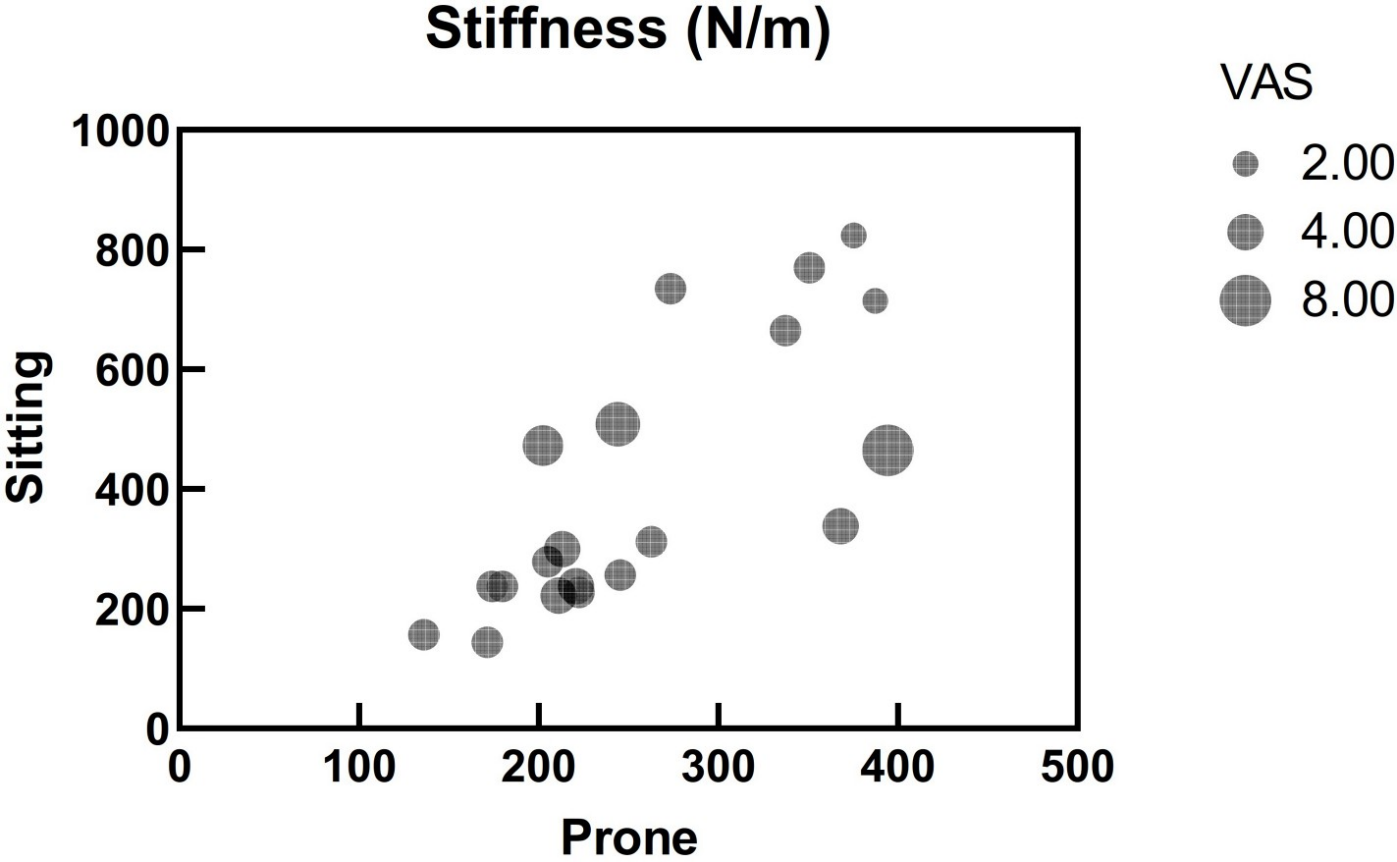
Bubble chart of relationships between visual analog scale (VAS) and erector spinae stiffness on the painful side in prone and sitting (*N* = 20).

Table 3 shows the relationships between VAS and erector spinae stiffness on the painful side. There was no significant correlation between the lumbar erector spinae muscle stiffness and VAS in different postures on the painful side (*r* = -0.078, *P* > .05 and *r* = -0.107, *P* > .654, respectively) (Fig 4).

**Table 3:** Spearman’s rank correlations between VAS and the erector spinae stiffness on painful side.

	Painful side	VAS	*r*	*P*
Prone	258.68±81.13	3.60±1.39	-0.078	.745
Sitting	404.65±222.46		-0.107	.654

VAS: visual analog scale.

## Discussion

The primary purpose of the study was to detect the intra- and inter-reliabilities of lumbar erector spinae stiffness by MyotonPRO among adults with CLBP in the prone position. Furthermore, we were able to compare erector spinae stiffness between painful and non-painful sides in different postures and the changes of stiffness from prone to sitting in painful and non-painful sides. This study also explored the correlation between intensity of pain and lumbar erector spinae stiffness among adults with CLBP. Overall reliability estimates were excellent with ICCs ranging from 0.88 to 0.99. The erector spinae stiffness on the painful sides was higher, with a marked increase in the sitting position when compared with the non-painful side. However, the intensity of pain among adults with CLBP was not associated with muscle stiffness of the lumbar erector spinae muscles.

In the present study, the ICCs (ICC = 0.99) were excellent for inter-rater reliability. In reference to intra-rater reliability, the ICCs (from 0.88 to 0.91) were good to excellent. The ICC values indicate the degree of consistency between the testing sessions. Within the highly reliable values, between-day reliability was lower than within-day reliability. A possible reason for the results may be attributed to the changes of muscle stiffness between days due to physical activity. Our reliability results are consistent with a previous study using MyotonPRO in young adults with CLBP, where ICC scores ranged between 0.86 and 0.96 [25]. In addition, Hu et al. [25] investigated intra-reliability for the parameters of muscle stiffness only at bilateral L_1_ to L_5_ levels in their study. Our study added to the comparisons of erector spinae stiffness between the painful side and the non-painful side in different postures, which may be one of the important causes of CLBP occurrence. In a previous study, Lohr et al. [10] demonstrated that the MyotonPRO was more reliable on repeated testing than tensiomyography on the muscle belly of the erector spinae muscle at level L_3_–L_4_ in healthy adults. Kelly et al. [16] reported that MyotonPRO reliability (ICC > 0.93) was better than SWE (ICC > 0.88) in the infraspinatus, erector spinae, and gastrocnemius muscles, and demonstrated the ability to discriminate the levels of muscle contraction.

The SEM was used to estimate how repeated measures tend to be distributed around the “true” score, and the MDC reflects the smallest amount of true change rather than measurement error inherent in the score [28]. For inter-rater reliability, all SEM% and MDC% were from 2.58% to 2.69% and from 7.14% to 7.46%, respectively. For intra-rater reliability, all SEM% and MDC% were from 6.93% to 9.14% and from 19.20% to 25.35%, respectively. Furthermore, the SEM% interprets the relative amount of measurement error and that of below 10% was arbitrarily considered to be small and acceptable [29]. An MDC% less than 10% can be considered excellent and one less 30% can be considered acceptable [29]. Of note, the MDC% values for intra-rater reliability were markedly superior to 10%.

Bland-Altman analyses were conducted to identify systematic bias and compare the 95% limits of agreement. A high agreement, providing a range of error that may relate to clinical acceptability, was shown with less systematic bias [30]. In other words, this means good agreement between the testing sessions when using the MyotonPRO to measure lumbar erector spinae stiffness in patients with CLBP [13]. Bland-Altman analyses have an advantage in that scatter plots can be used to visually interpret data from the observations of any outliers, bias, or relationship between variance in measures, size of the mean, and limits of agreement [31]. When all of the measurements included 0, Bland-Altman analysis indicated no systematic bias [30]. In our study, the 95% CI of the mean difference included 0, which confirmed good repeatability.

The results of this study suggest that participants with unilateral CLBP had more lumbar erector spinae stiffness on the painful side than on the non-painful side in sitting. The painful side is 13.31% larger than the non-painful side (404.65N/m and 357.13N/m, respectively). However, there was no significant difference in the lumbar erector spinae muscle stiffness in the prone position. Our results were in accordance with previous studies using SWE in patients with CLBP, where CLBP is associated with muscle stiffness of the lumbar multifidus muscle in young and middle-aged medical workers [22]. In a recent study, Hu et al. [25] reported that no significant difference was observed between the left and right side pooled paraspinal muscle stiffness among young adults with CLBP in the prone position (Left side = 280.9N/m and right side = 289.7N/m at L_4_ levels). However, this study did not distinguish between the painful side and the non-painful side. Compared with the prone posture, pain was primarily featured by the presence of a palpable taut band within the erector spinae muscle in a sitting posture. It may be that pain transmitted through the nerves negatively affects local soft tissues. In our previous study, Zhang et al. [32] demonstrated that the shear elastic modulus of the vastus lateralis muscle was increased by 26.5% in the subjects with patellar tendinopathy when compared with those of healthy controls. Kuo et al. [33] indicated that participants with chronic neck pain had a significantly stiffer trapezius muscle.

Interestingly, we demonstrated that the erector spinae stiffness was significantly increased from prone to sitting, especially on the painful side. Becker et al. [34] reported that the activity of the lumbar erector spinae was significantly higher in patients with CLBP from sit to stand, 30 seconds of standing, and climbing stairs, and significantly lower during static waist flexion compared with healthy controls. Chen et al. [35] found significantly higher passive biceps brachii muscle stiffness at full elbow extension compared to 30° of flexion by ultrasound SWE. The erector spinae stiffness in our study increased from prone to sitting, especially for the painful sides. Painful areas in the erector spinae muscle may be latent myofascial trigger points, which are sensitive spots where pain is only elicited in response to different kinds of stimulation (e.g., as from prone to sitting).

In our study, there was no significant correlation between the lumbar erector spinae muscle stiffness and pain intensity in different postures on the painful side (Fig 4). In contrast to the previous study, the location for stiffness measurement in the present study was identified on the palpable muscle belly one-finger breadth from the spinous process at level L_4_ rather than the painful region. For example, Zhang et al. [26] found that there was a significant relationship between tendon shear elastic modulus ratio (painful over non-painful tendon) and the intensity of pain in the painful region. In this study the shear wave elastography was used to quantify the stiffness of tendon within painful region.

The current research has a certain positive effect on guiding clinical practice. In a healthy state, the human musculoskeletal system is in a balanced state of left-right symmetry. When some bad postures appear, this balance is gradually broken and a morbid state appears. In our previous study of healthy people, there was no difference in the erector spinae stiffness on both sides in prone, sitting, and standing positions [20]. However, in this study, the stiffness of the erector spinae on the painful side was significantly higher than that on the non-painful side only in the sitting state for participants with unilateral CLBP. For the symmetry assessment of this situation, it is difficult to make accurate judgments only by the doctor’s palpation or posture observation. Furthermore, MyotonPRO can also be used to evaluate soft tissue stiffness before and after the intervention, such as paraffin therapy [36], dry needling [37], and massage [38]. In addition, MytonPRO has excellent reliability and portability, and the evaluation is not limited by personnel and venues. In the future, accurate assessment of soft tissue stiffness may be used as a basic clinical assessment method to better serve patients.

We realize that there are some limitations to this study. First, the measurement sites were defined at particular area of the muscle that doesn’t represent the condition of the whole muscle. In this study, we presumed that painful and non-painful sides within the same participants was considered as separate individuals. However, there could be pathological cross-effects from painful side. Second, the lumbar erector spinae muscles consist of multiple small muscles interspersed between verifying fascial planes. In our study, the measurement of muscle stiffness was acquired on the palpable muscle belly one-finger breadth from the spinous process at level L_4_ rather than the pain location. In addition, self-reported VAS score has certain subjectivity. We recognized that relationships between VAS and the erector spinae stiffness on painful side may be affected. Third, the muscle stiffness of the elders might be different with the youngers according to previous studies [39, 40]. The present study, the age distribution span of the participants was large and therefore, our findings could not be generalized to the specific population groups such as the elders and the youngers. Given the promising results of our study, an important next step is to further establish the efficacy of age and gender on muscle stiffness. Fourth, the 5-day interval in the intra-rater reliability, the changes in the participants’ condition may affect the changes in muscle stiffness and thus affect the results to a certain extent. Moreover, as a single indicator, VAS scores did not representative of all clinical symptoms of unilateral CLBP. Finally, there are some inherent limitations of the MyotonPRO device. For example, only the lumbar erector spinae stiffness was assessed in this study, as the MyotonPRO device cannot be used for the measurement of deep muscles located under layers of other tissues. Furthermore, it would seem that the thickness of subcutaneous fat may have an impact on the stiffness since the test is not directly on the muscle.

## Conclusions

In the current study, we indicated that the MyotonPRO device has excellent intra- and inter-rater reliabilities in quantifying the stiffness of the lumbar erector spinae muscles in patients with CLBP. Meanwhile, the erector spinae stiffness on the painful sides was higher with a marked increase in sitting when compared with the non-painful side. Hence, the MyotonPRO can be used in clinical assessment and research in patients with CLBP, although there was no significant correlation between the lumbar erector spinae muscle stiffness and pain intensity.

## Supporting information

S1 Data (DOCX)

S1 Checklist STROBE statement—checklist of items that should be included in reports of observational studies. (DOCX)

## Abbreviations

CLBP: Chronic lower back pain
VAS: Visual analog scale
ICC: Intraclass correlation coefficients
MDC: Minimal detectable change
EMG: Electromyography
MRE: Magnetic resonance elastography
SWE: Shear wave elastography
BMI: Body mass index
SD: Standard deviations
95%CI: 95% confidence interval
SEM: The standard error measurement
