# Supplementary material for: Quantifying the stiffness of lumbar erector spinae during different positions among participants with chronic low back pain
Source: PLoS One. 2022 Jun 24;17(6):e0270286. doi: 10.1371/journal.pone.0270286 (PMC9231815; doi:10.1371/journal.pone.0270286)
Supplement: S1 Data — (DOCX) [file pone.0270286.s001.docx]

**Research Data**

**Table1. Reliabilities data (Stiffness; N/m)**

**Table2. The stiffness of erector spinae in different positions (Stiffness; N/m)**

| **Table1. Reliabilities data (Stiffness; N/m)** | | | | | | |
| --- | --- | --- | --- | --- | --- | --- |
| **No.** | **LeftA1** | **LeftB** | **LeftA2** | **RightA1** | **RightB** | **RightA2** |
| **1** | 392 | 409.67 | 470.67 | 409 | 422.33 | 416 |
| **2** | 467.33 | 478 | 505.67 | 434 | 426.33 | 506.33 |
| **3** | 350.67 | 350 | 388.67 | 289.33 | 293.67 | 390.67 |
| **4** | 237.67 | 244.67 | 236 | 267.67 | 269 | 253.33 |
| **5** | 405.33 | 414 | 431.67 | 435.67 | 421.33 | 436.33 |
| **6** | 386.33 | 387.33 | 361.67 | 386 | 394.33 | 402.67 |
| **7** | 177 | 173.33 | 242.33 | 195.33 | 187.33 | 237 |
| **8** | 283.33 | 320 | 408.33 | 405.33 | 394 | 378.67 |
| **9** | 291.33 | 302.33 | 311.33 | 275.33 | 274.67 | 315.33 |
| **10** | 412.33 | 425.67 | 343 | 575.33 | 499.67 | 467.67 |

| **Table2. The stiffness of erector spinae in different positions (Stiffness; N/m)** | | | | | |
| --- | --- | --- | --- | --- | --- |
| **No.** | **The painful sides in the sitting position** | **The non-painful sides in the sitting position** | **The painful sides in the prone position** | **The non-painful sides in the prone position** | **VAS** |
| **1** | 734.33 | 629.67 | 273.33 | 381 | 3 |
| **2** | 769.33 | 736 | 350.67 | 342.33 | 3 |
| **3** | 823.33 | 751 | 375.33 | 346.67 | 2 |
| **4** | 664.33 | 610.67 | 337.33 | 303.33 | 3 |
| **5** | 337.67 | 352.33 | 368 | 349.33 | 4 |
| **6** | 299.33 | 233 | 213 | 219.33 | 4 |
| **7** | 278.33 | 258.33 | 205 | 198.67 | 3 |
| **8** | 464.33 | 402.33 | 394.33 | 358 | 8 |
| **9** | 472.33 | 357.33 | 202.33 | 279 | 5 |
| **10** | 714 | 484.33 | 387.33 | 325.67 | 2 |
| **11** | 237 | 227.67 | 174 | 138.67 | 3 |
| **12** | 237.33 | 220.33 | 220.67 | 210 | 4 |
| **13** | 311.67 | 303.33 | 262.67 | 265.67 | 3 |
| **14** | 227 | 239 | 222.33 | 225.33 | 3 |
| **15** | 237 | 239.33 | 179.67 | 196 | 3 |
| **16** | 256 | 239.67 | 245.33 | 226.33 | 3 |
| **17** | 221.67 | 217.33 | 211 | 217.67 | 4 |
| **18** | 156.33 | 129.33 | 136 | 175.67 | 3 |
| **19** | 508 | 370 | 244 | 265 | 6 |
| **20** | 143.67 | 141.67 | 171.33 | 120.33 | 3 |
